# Withaferin A: From Ancient Remedy to Potential Drug Candidate

**DOI:** 10.3390/molecules26247696

**Published:** 2021-12-20

**Authors:** Tahira Sultana, Mohammad K. Okla, Madiha Ahmed, Nosheen Akhtar, Abdulrahman Al-Hashimi, Hamada Abdelgawad, Ihsan-ul- Haq

**Affiliations:** 1 Department of Pharmacy, Faculty of Biological Sciences, Quaid-i-Azam University, Islamabad 45320, Pakistan; tahira.sultana69@gmail.com; 2 Botany and Microbiology Department, College of Science, King Saud University, Riyadh 11451, Saudi Arabia; okla103@yahoo.com (M.K.O.); al-ghamd@gmail.com (A.A.-H.); 3 Shifa College of Pharmaceutical Sciences, Shifa Tameer-e-Millat University, Islamabad 44000, Pakistan; 4 Department of Biological Sciences, National University of Medical Sciences, Rawalpindi 43600, Pakistan; nosheenakhtar@numspak.edu.pk; 5 Integrated Molecular Plant Physiology Research, Department of Biology, University of Antwerp, 2020 Antwerpen, Belgium; hamada.abdelgawad@uantwerpen.be

**Keywords:** anticancer, antitumor, antiinflammatory, antiherpes virus, antileishminial, *Withania somnifera*, withaferin A

## Abstract

Withaferin A (WA) is a pivotal withanolide that has conquered a conspicuous place in research, owning to its multidimensional biological properties. It is an abundant constituent in *Withania somnifera* Dunal. (Ashwagandha, WS) that is one of the prehistoric pivotal remedies in Ayurveda. This article reviews the literature about the pharmacological profile of WA with special emphasis on its anticancer aspect. We reviewed research publications concerning WA through four databases and provided a descriptive analysis of literature without statistical or qualitative analysis. WA has been found as an effective remedy with multifaceted mechanisms and a broad spectrum of pharmacological profiles. It has anticancer, anti-inflammatory, antiherpetic, antifibrotic, antiplatelet, profibrinolytic, immunosuppressive, antipigmentation, antileishmanial, and healing potentials. Evidence for wide pharmacological actions of WA has been established by both in vivo and in vitro studies. Further, the scientific literature accentuates the role of WA harboring a variable therapeutic spectrum for integrative cancer chemoprevention and cure. WA is a modern drug from traditional medicine that is necessary to be advanced to clinical trials for advocating its utility as a commercial drug.

## 1. Introduction

Withanolides are steroidal lactones, with the ergostane skeleton having diverse biological and pharmacological activities [1]. Withanolides specifically exist in the plant genera of the *Solanaceae* family including *Withania, Physalis, Datura, Nicandra, Dunalia, Lyerium, Tubocapsicus* and *Jaborosa*. These have also been isolated from *Lamiaceae, Taccaceae* and *Fabaceae* families and some marine sources [1]. Among the withanolide constituents isolated to date, withaferin A (WA) has attained the interest of the pharmacologists, clinical pharmacists, and chemists owing to its wide-ranging therapeutic potential. Withaferin A is one of the withanolides that is of profound medicinal importance. It is abundantly found in the plant *Withania somnifera* (WS) Dunal, which is an important Ayurvedic remedy, recognized with the common name of Ashwagandha. WS has been utilized as a primary component in various traditional formulations for addressing multiple health issues as well as to confer them more effectively. This stupendous plant has many pharmacologically well-established activities such as anti-inflammatory, antistress, antitumor, antioxidant, radiosensitizing, hepatoprotective, anticonvulsant, immunomodulatory, antiproliferative, cardioprotective, thyroid stimulating, hypoglycemic, diuretic, and hypercholesterolemic properties. It is also employed to cure musculoskeletal disorders such as arthritis and rheumatism as well as neurodegenerative disorders. Moreover, it is used as a tonic to boost energy, improve longevity, and also prevents diseases in pregnant females, geriatrics, and athletes. In Ayurveda, Ashwagandha and ginseng are believed to be adaptogens due to their adaptive reactions to illness, responsiveness to many unrelated diseases, and production of nonspecific augmented resistance to adverse effects of medicines [2]. One of the most promising hallmarks of WS is its anticancer and chemo-preventive potential, which has been affirmed from multiple studies [3]. The role of WS as anticancer drug is further accentuated by studies demonstrating inhibition of transplanted tumors in mice [4]. Administration of WS root extracts (150 mg/kg/day) brought about a 21–23% decrease in tumor burden and multiplicity of mammary tumor, induced by methyl nitrosourea in rat models [5]. Chemotherapy induced toxicity and fatigue were also reported to be alleviated in breast cancer patients [6]. Anticancer effect of WS is credited to the presence of withanolides [7]. Several other plants species are reported to possess this important withanolide. The most common medicinal plants bearing a significant amount of WA are listed in Table 1.

Molecular and pharmacological investigations on WA have asserted that the beneficial effects of WS are associated with withanolides [8]. Considering the significance of WA, this review is designed to narrate important pharmacological activities and associated molecular mechanisms of WA with special emphasis on anticancer profiles. 

**Table 1:** Some medicinal plants species containing withaferin A.

Plant	Common Name	Family	Part Used	Reference
*Acnistus arborescens*	Gallinero	Solanaceae	Leaves	[9]
*Acnistus breviflorus*	--	Solanaceae	Excised Leaves	[10]
*Ajuga bracteosa*	Kauri booti	Lamiaceae	Whole plant	[11]
*Physalis longifolia*	Common ground cherry	Solanaceae	Aerial part	[12]
*Vassobia breviflora*	--	Solanaceae	Aerial part	[13,14]
*Withania aristata*	--	Solanaceae	Leaves	[15,16]
*Withania coagulans*	Paneerdoda/Habbul	Solanaceae	Root culture	[17]
*Withania obtusifolia*	--	Solanaceae	Leaves	[18]
*Withania somnifera*	Ashwagandha	Solanaceae	Fruits	[19]

## 2. Chemistry of Withaferin A

**Figure 1:**
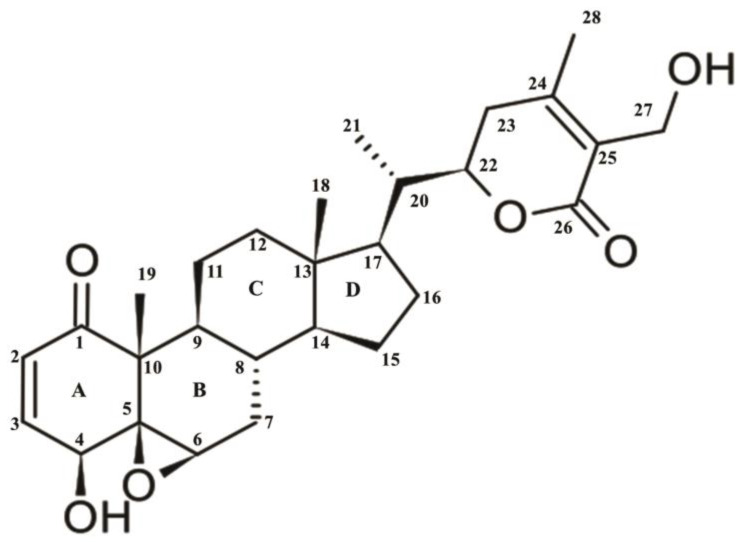
Structure of withaferin A.

Withaferin A [(4β,5β,6β,22*R*)-4,27-dihydroxy-5,6:22,26-diepoxyergosta-2,24-diene-1,26-dione] is a 28 carbon containing withanolide having ergostane framework and a δ-lactone [20,21]. According to the qualitative structure-activity relationship (QSAR), WA has two hydrogen bond donors and six hydrogen bond acceptor groups that make it highly reactive. The three site of WA including ketone containing unsaturated ring-A at C_2=3,_ epoxide structure at C_5_ in ring-B and unsaturated lactone ring are the hosts for nucleophiles. For example, cysteine sulfhydryl groups of proteins target lactone or epoxide of WA (Figure 1). It has been verified that double bond at C_2=3_ and epoxide at C_5-6_ are accountable for the cytotoxicity of WA [22,23]. 

The research for the exploration of novel therapeutic agents is facilitated mainly by the automated screening process or high throughput screening (HTS). These strategies play an important role in the selection of active entities among the thousands of synthesized or naturally derived products (using a battery of bioassays) [24]. The HTS strategy is accompanied with the computational filter screening of the compounds on the basis of chemical structures. The main purpose of this process is to rule out the compounds having undesired properties, as well as the chances of false positive results which can hinder the marketing of those compounds as drugs. These compounds are termed as PAINS (pan-assay interference compounds), which are capable of forming aggregates with the proteins and/or interfere in bioassays to promote false positive results [24]. Although WA has not been studied in this regard, certain other steroidal withanolides (bearing a trans-hyndridane dehydro-d-lactone scaffold) have stereoselectively been synthesized using the biology-inspired synthesis (BIOS) approach and using three key intermediates with different functional groups. The synthesized compound showed tremendous inhibition of the Hedgehog signaling pathway (which target the smoothened proteins) [25].

## 3. Pharmacological Profile of WA

This compound is well known for its wide array of therapeutic activities, which has been discussed in the various sections of this review.

### 3.1. Anticancer and Cancer Chemopreventive Effect

**Figure 2:**
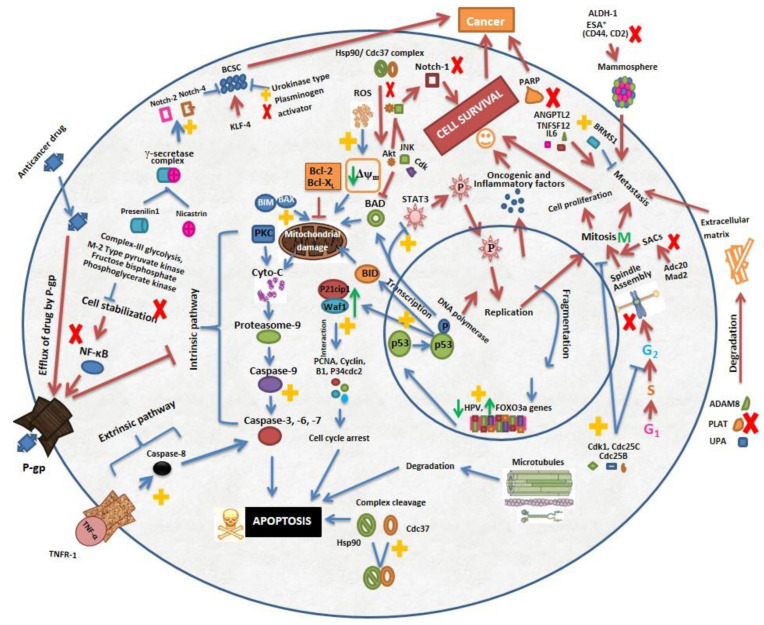
Summarized and diagrammatic representation of mechanism of anticancer activity of Withaferin A. Withaferin A targets multiple cell proliferation and apoptosis pathways. Major apoptosis pathways include activation of Notch-4, Bax, FOXO3a, and extrinsic and intrinsic apoptosis pathways, p53 induction, p21cip1/waf1 activation, Hsp90/Cdc37 complex cleavage, ROS production, and microtubule cleavage. It also inhibits cancer proliferation by blocking Notch-1, Atk/JNK, PARP, STAT3 and inhibit metastasis by enhancing BRMS1 expression and decreasing expression of ANGPTL2, TNFSF12, IL6, ALDH-1, CD44, CD2, and extracellular matrix degrading protein ADAM8, PLAT, and UPA. It causes mitotic arrest by activating Cdk1, Cdc25C, Cdc25B, PCNA, and p34cdc2 protein and by inhibiting Adc20, MAD2, and KLF-4 proteins. Withaferin A also reduces NF-κB pathways, p-glycoprotein and expression of HPV oncoproteins thereby mediating p53 and pRb associated apoptosis. ↑ and ↓ indicates increase and decrease in expression level, respectively. Red and blue arrows (and) show pathways associated with cell proliferation and apoptosis respectively. Symbols 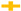 and 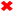 shows pathways stimulated and blocked respectively by WA.

Cancer is a multimode disease that corrupts physiological mechanisms and invades healthy cells. In order to improve the prognosis and survival of cancer patients, highly effective strategies are required. WA is instrumental in combating cancer via targeting diverse molecular markers. It has been established from flow cytometry and real time cell-proliferation assays that WA targets various processes related to cell death, cell proliferation and cell cycle. Heat shock proteins (Hsp) are specific types of molecules responsible for the aggravation and stability of oncogenes. Hsp90 (90 kDa heat shock protein) is one of these chaperons that interacts with co-chaperon Cdc37 for its normal function and stabilizes oncogenes for cell survival. Disassembly of this complex disrupts chaperon-protein interaction, consequently leading to apoptosis of tumor cells. WA exerts plasticity and disruption of this complex disabling survival of cancer cells [26]. Similar actions were also documented in another study that reported WA mediated inhibition of Hsp90 followed by degradation of client proteins including Akt, Cdk4, and glucocorticoid receptors (GR) [15]. This action was reversed by proteasomal inhibitors such as MG132. A co-immunoprecipitation assay revealed that WA at 10 mM concentration disrupted Hsp90/Cdc37 complex but it did not block ATP binding site of Hsp90 indicating that blockage of Hsp90/client protein by WA is ATP independent [27]. Paradoxically, upon screening of 80,000 compounds (natural and synthetic) WA was found to be the only inducer of heat shock (HS) response. This effect was evaluated by the expression of the HS factor controlling gene. The activation of such response by WA was expected due to the cytoprotective effect [28]. On the contrary, some reports suggested that anticancer effects are associated with inhibition of Hsp (Table 2). This controversial behavior might be the consequence of administered WA concentration and the cell type used. Cancerous cells activate the HS factor to control transcriptional responses, which enables cell survival in stressful conditions. WA blocked these responses and enhanced stress capacity resulting in cellular death [29]. The describing mechanism of the anticancer activity of WA and its mode of action are presented in Figure 2.

The anticancer effect of WA is also mediated by autophagy, which is an evolutionary physiological process of macromolecules for bulk degradation. It was demonstrated that autophagy was induced in spontaneously immortalized and non-tumorigenic normal human mammary epithelial cell line MCF-10A and human breast cancer cell lines MDA-MB-231 and MCF-7 when they were exposed to WA. WA treatment to MDA-MB-231 xenografts also revealed the markers for autophagy [30]. Furthermore, WA greatly enhanced the sensitivity of doxorubicin, a potent anticancer drug both in vivo and in vitro [31].

WA suppressed MDA-MB-231 invasiveness by suppressing the expression of extracellular matrix degrading proteases such as uPA, ADAM8 and PLAT at the transcriptional level. Pro-inflammatory mediator genes (TNFSF12, IL6, ANGPTL2, and CSFIR) responsible for metastasis promotion and cell adhesion molecules were also inhibited. Administration of WA has also been associated with the variations in the expression of breast cancer metastasis suppressor gene 1 (BRMS1). These changes convened antimetastatic and antiinvasive effects of WA [32]. Metastasis was inhibited in breast cancer by WA via vimentin (motility inducing substance for cells) inhibition and induction of vimentin ser56 phosphorylation at a dose of 500 nM that was non-toxic to normal cells. In fact, WA caused accumulation of vimentin around the perinuclear wall followed by depolymerization of vimentin, causing rapid death of cancer cells [33]. 

Metastasis in breast cancer cells is also associated with Notch activation. Presenilin1 and nicastrin form γ-secretase complex that activates Notch 4 by cleaving it from Notch 2. Activated Notch 2 and 4 caused the inhibitory effect of WA on breast cancer cell migration and metastasis [34]. Besides, WA cleaved and inhibited Notch 1 that interacts with the pro-survival components Akt/NF-κB/Bcl-2 [35]. Thus, WA targets different pathways in cancer cells to inhibit their proliferation and survival.

#### 3.1.1. Apoptosis Induction in Cancer Cells

Apoptosis is the primary mechanism to clear cancer cells from the body. WA associated apoptosis in tumor cells lessen cancer progression and survival. P-glycoproteins (P-gp) are the drug efflux proteins and are also blockers for caspase dependent apoptotic pathway stimulated by hyper-active NF-κB, AP1, and Nrf2 (Table 2). WA target this NF-κB transcription factor instigating chemosensitivity in cells with overexpressed P-gp and trigger tumor cell apoptosis [36]. In a study, the effect of WA on doxorubicin sensitive and resistant cells (K562 and K562/Adr, respectively) was determined and it was found that WA disabled attenuated caspase activation and apoptosis in K562/Adr cells. Moreover, not only the protein levels of Bcl2 and P-Bad were decreased, but the cytoskeletal tubulin protein levels were also reduced along with cleavage of potent poly-(ADP-ribose)-polymerase (PARP), caspase3 activation and apoptosis by thiol-oxidation pathway [36]. 

Another report revealed that WA caused cytotoxicity in MCF-7 cells by apoptosis as well as dose dependent arrest in G2 and M phases of mitosis causing decreased cancer growth and viability. Apoptosis in MCF-7 was induced by down-regulation of the expression of heat shock factor 1 (HSF1), RET (rearranged during transfection of tyrosine kinase), estrogen receptor α (ERα) and up-regulated expression of phospho-p38 MAPK (phosphorylated p38 mitogen-activated protein kinase), p38 stress activated protein kinase (SAPK), p53 and p21 proteins via inhibition of proteasome chymotryptic activity. ERα down-regulation is associated with proteasome-dependent degradation of ERα [37,38,39]. WA has also been reported to suppress many genes that regulate metastasis and cell growth such as c-myc as well as CARP-1/CCAR-1, a transducer for signaling cell growth. This compound concomitantly inhibited malignant pleural mesothelioma growth (MPM) by targeting multiple pathways (e.g., blocking the activity of proteasome). Stimulation of apoptosis thus holds significance as an anti-MPM agent [38].

WA prompts the production of reactive oxygen species (ROS) to induce oxidative stress over the mitochondrial membrane and dissipate its potential (Δψ_m_) with a consequent release of cytochrome C. This results in the eventual generation of caspase such as proteases and oligonucleosomal DNA fragmentation. It also causes induction of cell nuclei to produce apoptotic factor and translocation of proapoptotic protein Bcl2-associated X protein (BAX) to mitochondria. Parallel to these events is the cleavage of PARP and activation of caspases 3 and 9. It also significantly activates the extrinsic pathway, which was verified by dose dependent activation of caspase-8 via TNFR-1 overexpression. These findings demonstrated that WA triggered ROS generation and loss of normal mitochondrial function in cancerous cells thus responsible for stimulation of mitochondrial dependent and independent apoptotic pathways [40,41].

WA also augments the susceptibility of cancer cells towards apoptosis by dissipating Bcl-2/Bax and Bcl-2/Bim ratios [41]. Additional crucial targets of WA for apoptosis are JNK and Akt (a cell surviving protein) signal pathways. Activated (phosphorylated) Akt regulates cell survival in cancer cells. It was demonstrated that treatment with WA induced apoptosis by dephosphorylation of Akt through NF-κB [42]. Apoptosis was also induced partly by c-Met/Akt suppression and Raf-1 signaling activation as observed in intraocular melanoma cells [43]. WA also induced prostate apoptosis responsive-4 (PAR-4) gene, which ubiquitously caused p53 induction and PTEN independent and cancer specific apoptosis [44]. 

Human papilloma virus (HPV) causes cervical cancer by activating E6 and E7 oncoproteins, which are responsible for the suppression of p53 and pRb proteins, respectively. WA exhibited an inhibitory effect on cervical cancer cell (CaSKi) proliferation with IC_50_ of 0.45 ± 0.05 µM. It also induced accumulation of p53 and up-regulation of p21 (cip1/waf1) by down-regulating the expression of E6 and E7 oncoproteins. Furthermore, it enhanced p21 interaction with proliferating cell nuclear antigen (PCNA). This led to G2/M cell cycle arrest, decreased levels of STAT3 and its phosphorylation at Tyr705 and Ser727 and alteration of cyclin B1 and p34 (cdc2) levels [45]. All of these events proceeded towards apoptosis of cervical cancer cells (Table 2).

WA-induced apoptosis is also mediated by disabling angiogenesis in the tumor. Vimentin, an intermediate filament (IF) is essential for angiogenesis and cancer growth. Aggregation of this protein disrupts the cytoskeleton and inhibits angiogenesis. It has been established that WA covalently binds with vimentin via modification in its cysteine residue and induced aggregation of vimentin with F-actin both in vitro and in vivo. This cytoskeleton perturbing the activity of WA inhibits neovascularization and causes endothelial cell apoptosis [46].

Mitotic delay is another source for apoptosis and cancer prevention. WA also destabilizes chromosomes by delaying mitotic exit as well as blocking the function of spindle assembly checkpoints (SAC). Moreover, apoptosis is induced by the degradation of Mad2 and Adc20 (two important components of SAC) by proteosomal enzymes [47,48]. It was observed that G2 and M phase arrest is associated with cyclin dependent kinase 1 (Cdk1), cell division cycle 25C (Cdc25C) and Cdc25B proteins, which led to the accumulation of inactive Cdk1 (a phosphorylated form of Cdk1) [48]. All of these studies revealed the molecular mechanisms of WA induced apoptosis in cancer cells, ranking it as a potent anticancer agent. 

**Table 2:** Summary of studies on effect of withaferin A on different cell lines.

**Cell Line/Model**	**Study Type**	**Mechanism of Action**	**Effect**	**Dose**	**References**
		**Anticancer**			
Breast cancer cells MCF-7	In vitro	Down-regulation: ER-α, RET tyrosine kinase, HSF1 Up-regulation: phospho-p38 MAPK, p53 and p21	Apoptosis	576 nM	[39]
MDA-MB-231 (estrogen independent) MCF-7 (estrogen-responsive)	In vitro	G2 and M phase cell cycle arrest	Apoptosis	-	[48]
Human breast cancer cells	In vitro	Notch2 and Notch4 activation	Apoptosis	-	[35]
Breast cancer cells (MDA-MB-231 and MCF-7)	In vitro	FOXO3a- and Bim activation	Apoptosis	-	[49]
Breast cancer cells (MDA-MB-231 and MCF-7)	In vitro	Activation of signal transducer and activator of transcription-3	Apoptosis	-	[50]
Breast cancer cells (MDA-MB-231)	In vitro	NF-κB inhibition by IL6 inhibition	Apoptosis	-	[51]
Doxorubicin-sensitive human leukemic (K562) and -resistant K562/Adr cells,	In vitro	IL6, IL8, A1,MCP1, A20, cyclinD1, VEGF, MDR1 genes inhibition	Apoptosis	-	[36]
Multiple myeloma cell line U266)	In vitro	NF-κB inhibition	Apoptosis	-	[52]
Human myeloid leukemia (HL-60)	In vitro	ROS generation and mitochondrial dysfunction	Apoptosis	-	[53]
Myeloid leukemia (KBM-5) cells	In vitro	Inhibition of NF-κB	Apoptosis	-	[7]
T-cell lymphoma (HUT-78), Human myeloid leukemia cells (HL60)	In vitro	Inhibition of NF-κB	Apoptosis	-	[40]
human leukemic monocyte lymphoma (U937) cells	In vitro	Cleavage of PARP, activation of caspase-3 and Bcl-2 down-regulation	Apoptosis	-	[42]
Human melanoma cells	In vitro	ROS generation and Bcl-2 down-regulation	Apoptosis	-	[41]
Uveal melanoma cells	In vitro	Suppression of Akt and c-MET activation	Apoptosis	-	[43]
Cervical cancer cells (CaSki)	In vitro	Induces p53, repression of HPV oncogenes, up-regulation of tumor suppressor proteins	Apoptosis	0.45 ± 0.05 mM	[45]
Human cervix adenocarcinoma cell line (Hela)	In vitro	Inhibition of NF-κB		-	[54]
Colon carcinoma cell lines (HCT116, SW480, SW620)	In vitro	NF-κB inhibition, SAC dysfunction by degrading Mad2 and Cdc20 proteins and hence mitotic delay	Arrest proliferation	-	[47]
Colon carcinoma cell line (SW480)	In vitro	Inhibition of NF-κB	Arrest proliferation	-	[55]
Immortalized Cystic fibrosis airway cell line (KKLEB), Human embryonic kidney (HEK) cells	In vitro	Inhibition of NF-κB by IL8 gene	Arrest proliferation	-	[56]
Fibrosarcoma (L929)	In vitro	IL6, RANTES, IkBa gene inhibition leads to inhibition of NF-κB	-	-	[53]
**Human umbilical vein endothelial cells** (HUVEC)	In vitro	IL6, TNF-αgene suppression	Arrest proliferation	-	[57]
Adipocyte cell line (3T3-L1)	In vitro	Increased ERK1/2 phosphorylation and altered Bax and Bcl2 protein expression,	Apoptosis	1–25 μM	[58]
		decrease lipid accumulation, expression of peroxisome proliferator-activated receptor γ, CCAAT/enhancer binding protein α and adipocyte fatty acid binding protein	Inhibits adipogenesis	0.1–1 μM	
		**Cancer Prevention**			
DMBA induced oral cancer in Syrian golden hamsters	In vivo	Decrease lipid peroxidation, enhance antioxidant defense	Inhibits oral cancer (100%)	20 mg/kg, oral 3 times/week	[59]
Breast cancer in transgenic mouse mammary tumor virus-neu (MMTV-neu)	In vivo	Inhibits macroscopic and microscopic tumor burden (promotes apoptosis, inhibits complex III and glycolysis)	Apoptosis	100 μg/mouse, i.p., 3 times/week	[30]
DMBA induced squamous cell carcinoma in buccal cavity of Syrian Golden Hamsters	In vivo	Prevent alterations of p53 and Bcl-2 expressions	Inhibit tumor proliferation	-	[60]
		**Transplanted Tumors Inhibition**			
Ascites sarcoma (S-180) xenografts	In vivo	Vacuolization of cytoplasm, distention or dissolution of mitochondrial cristae, disruption of microtubules of mitotic spindles	Inhibit tumor growth and promotes survival	30 mg/kg, i.p.	[61]
Prostate cancer (PC-3) xenografts	In vivo	Promotes Par-4 and apoptosis	Inhibits tumor growth	5 mg/kg, intra-tumor, 5 days per week	[44]
Medullary thyroid cancer (DRO81-1) xenografts	In vivo	Decreases Total and phospho-RET	Inhibits tumor growth	8 mg/kg, i.p., daily	[62]
Fibrosarcoma (SKLMS-1), leiomyosarcoma (HT-1080) xenografts	In vivo	Inhibits PCNA and CD31, enhance apoptosis	Inhibits tumor growth	2 mg/kg, i.p., daily	[63]
Mouse breast cancer (4T1) xenografts	In vivo	Activates Ser56 phosphorylation of vimentin	Inhibits tumor growth	2 and 4 mg/kg, i.p., every other day	[33]
Mesothelioma (AB12) xenografts	In vivo	Inhibits proteasomal chymotrypsin-like activity	Inhibits tumor growth	5 mg/kg, i.p., daily	[38]
Pancreatic cancer (Panc-1) xenografts	In vivo	Inhibit Hsp-90 and degrade Akt, Cdk4 and glucocorticoid receptor	Inhibits tumor growth	3 and 6 mg/kg, i.p., 2 times per week	[27]
		**Anti-inflammatory effect**			
CF related inflammatory cells	In vitro	Inhibition of NF-κB	-	-	[56]
Human Islet Cells	In vitro	Inhibition of cytokines and TNF-α	-	1 μg/mL	[64]
Macrophage cell line (RAW 264.7)	In vitro	Inhibition of NF-κB and iNOS, Akt and COX-2 expression	-	-	[42]

**Abbreviations:** 7, 12−Dimethylbenz [A] anthracene (DMBA), Adipocytes cell line (3T3−L1), Ascites Sarcoma cells (S−18), Breast cancer cells (MCF−7, MDA−MB−231), Breast Cancer Metastasis Suppressor Gene 1 (BRMS1), Breast Cancer Stem cells (BCSC), Cancer Stem cells (CSC), Cervical Cancer cells (CASKI), Chicken B−Lymphocyte cell line (DT40), Colon Carcinoma cell lines (HCT116, SW480, SW620), Cystic Fibrosis Airway cell line (KKLEB), Fibrosarcoma cell lines (L929, SKLMS−1), Human Anaplastic Thyroid Cancer cell line (SW1736), Human Cervix Adenocarcinoma cell line (Hela), Human Embryonic Kidney cells (HEK), Human Leukemic cell line (K562), Human Leukemic Monocyte Lymphoma cells (U937), Human Myeloid Leukemia (HL−60), Human Papillary Thyroid Cancer cell line (BCPAP), Human Umbilical Vein Endothelial cells (HUVEC), Leiomyosarcoma (HT−1080), Macrophage cell line (RAW 264.7), Medullary Thyroid Cancer cells (DR081−1), Mesothelioma cell line (AB12), Mouse Breast Cancer cell line (4T1), Multiple Myeloma cell line(U266), Myeloid Leukemia cell line (KBM−5), Normal Human Mammary Epithelial cell line (MCF−10A), Pancreatic Cancer cells (PANC−1), Prostate Cancer cells (PC−3), T−Cell Lymphoma cell line (HUT−78).

#### 3.1.2. Antitumor Induction Activity

WA also exhibits its protective role against tumor development induced by some carcinogens. In a study, the hamster’s oral cavity was painted with 7, 12-dimethylbenz(a) anthracene (DMBA) to induce oral cancer. Among two groups, one was administered DMBA only and the second group was also given oral WA. First group showed signs and symptoms of squamous cell carcinoma along with alterations in the expression of p53 and Bcl-2 proteins. On the other hand, complete tumor inhibition and absence of alterations were observed in the case of WA treated group [60]. Another study on transgenic mouse mammary tumor virus-neu (MMTV-neu) model demonstrated that WA in a dose-dependent manner, inhibited the mammary cancer development by suppressing the breast cancer stem cells (BCSC) [30]. When WA was administered to MMTV-neu mice model at 100 µg/mouse dose thrice a week, the mammary tumor size, microscopic tumor area and occurrence of pulmonary metastasis were significantly decreased. The basis of these preventive activities is apoptosis, complex-III activity inhibition, and decreased glycolysis intermediates. Additionally, it down-regulated glycolysis related proteins (e.g., M2 type pyruvate kinase, phosphoglycerate kinase, and fructose bisphosphate A isoform 2) [30]. WA repressed epithelial specific antigen positive (ESA^+^) fractions/CD44^high^ and CD2^low^ as well as aldehyde dehydrogenase 1 (ALDH1). Mechanistically, it was found that WA conferred BCSC inhibition by Notch 4 activation as well as overexpression of urokinase type plasminogen activator. Furthermore, WA inactivated B-cell specific Moloney murine leukemia virus insertion region 1 (Bmi-1) whose abnormal expression led to the partial protection against the activity of ALDH1. Moreover, it also inhibited Kruppler-like factor (KLF-4) and its knockdown augmented BCSC inhibition [65].

#### 3.1.3. Sensitization to Chemotherapy

WA augments sensitization of tumor cells toward radiations as well as enhances cytotoxic effects of drugs to inhibit tumor progression [66]. In one report radiation resistant B16F1 mouse melanoma cells were studied with radio-sensitizing WA treatment in vivo. WA alone (10–60 mg/kg) in a dose dependent manner delayed tumor growth and volume expansion. On the other hand, a dose of 30–50 mg/kg followed by irradiation of 30 Gy γ-rays significantly enhanced the localized tumor response. Best results were observed when WA was injected intraperitoneally to test models, 1 h before radiation therapy. Although, complete tumor cure was not observed but the study emphasized that melanoma must first be treated with WA before radiation therapy to enhance its efficacy [67]. WA also exerts radio-sensitization by inhibiting DNA repair. To evaluate this concept, exponentially growing chicken B-lymphocyte cell line DT40 and its genetically modified forms having DNA repair gene mutations either in a single gene (homozygous Rad54-/- or Ku70-/-) or in double genes (Ku70-/- with Rad54-/-) were treated with 5 mM concentration of WA. This treatment was followed by X-ray irradiation of different intensities. Afterwards, the cell survival analysis indicated that WA sufficiently reduced survival of DT40 cells and repaired deficient cells with either Ku70-/- mutation alone or both Ku70-/- and Rad54-/- mutations [68].

Combined chemotherapy with carboplatin and paclitaxel followed by cytoreductive therapy is currently entailed in ovarian cancer treatment. This strategy is non responsive in some cases due to the relapse of cancer due to the resistance of cancer stem cells (CSCs) towards platinum. WA when used alone or in combination with cisplatin (platinum containing anticancer drug) targeted putative CSCs. This treatment resulted in 70-80% tumor growth reduction and completely inhibited metastasis. Western blot and histochemical analysis revealed that WA (2 mg/kg) eliminated CSCs markers as CD44, CD24, CD34, CD117, and Oct4 and down-regulated genes such as Hes1, Notch1and Hey1. On the contrary, the group which was administered cisplatin only, had increased Notch1 and other CSCs markers. It indicated relapse of cancer in the group with cisplatin alone and also explains reoccurrence in carboplatin and paclitaxel treated patients. Since WA alone or in combination with cisplatin decreased these markers; therefore, it is efficacious therapy for ovarian cancer [69,70]. Ionization radiations (IR) induced apoptosis in human lymphoma U937 was enhanced by WA with the associated cleavage of PARP, activation of caspase-3 and antiapoptotic protein Bcl-2 down-regulation [42]. The same results were observed in another study where the Caki cells were used [71]. 

Sorafenib (SN) is a multikinase targeting anticancer drug with enhanced toxicity. WA in combination with SN synergistically enhanced the treatment efficacy at sub-toxic doses. The molecular mechanisms involved were PARP cleavage, activation of caspase-3 and inhibition of BRAF/Raf-1. The evaluation was carried out by using human papillary (BCPAP) and anaplastic (SW1736) thyroid cancer cell lines. Two groups were provided with WA and SN each and the third group with both drugs in combination. IC_50_ were 6.3 (SN), 0.155 (WA), and 0.055 µmol/L (combination) for three groups in BCPAP and 7.6 (SN), 2.5 (WA), and 1.4 µmol/L (combination) in SW1736 cells. Flow cytometry showed an improvement of 89% in apoptosis, when the combination was employed [72]. This substantiates the effectiveness of WA both alone and in combination with standard anticancer drugs.

#### 3.1.4. Cancer Associated Anti-Inflammatory Effects

Inflammation is an essential component of a neoplastic process. A wide range of cancers arises from chronic inflammation at the site of cancer. For example, there is an increased risk of colon cancer in patients with uncontrolled Crohn’s disease and ulcerative colitis. Tumor cells are largely orchestrated by inflammatory cells that foster metastasis, proliferation and survival [73]. Withaferin A is a potent anti-inflammatory agent bearing capacity to combat cancer associated inflammation. By targeting molecular markers of inflammation, it can prevent both cancer initiation and progression. 

NF-κB transcription factor plays an important role in the pathways that lead to inflammation, angiogenesis, metastasis, antiapoptosis, and multidrug resistance [74,75]. Irregularity in NF-κB activity leads to prolonged inflammation and ultimately cancer. Several reports demonstrated that WA inhibits NF-κB transcription. The molecular mechanism for its inhibitory effect was previously studied [53], which demonstrated the inhibitory effect of WA, by interacting with IKK (I-κB kinase); a vital component for the translocation of NF-κB and its signaling pathways. The IKK complex is composed of two units, namely NEMO (NF-κB Essential Modulator) and IKKβ. WA was found to interact with both components by occupying the active site of IKKβ thus preventing its interaction with NEMO. Molecular studies revealed that Cys179 makes hydrogen bonding between WA and IKKβ to make stable interaction by accurately fitting WA in the groove of IKKβ. Mutation in Cys179 of IKKβ causes loss of interaction with WA [53]. The interaction of NEMO with WA was also studied via semi-flexible docking analysis, which depicted strong molecular binding with NEMO chain providing steric as well as a thermodynamic hindrance for incoming IKKβ component. Both studies provided significant evidence of the inactivation of NF-κB by disruption of NEMO/IKKβ complex [76]. 

Inhibition of NF-κB is a hallmark of anti-inflammatory activity of WA [76,77]. In a study, inflammation was induced by monosodium urate crystals to mimic model of gouty arthritis. Withaferin A (30 mg/kg) when administered through the intraperitoneal route reduced the values of lysosomal enzymes, lipid peroxidation, paw volume and TNF-α in mice near to normal level. The levels of glucuronidase and dehydrogenase were also found to be reduced by WA in polymorphonuclear leucocytes incubated with monosodium urate crystals [78]. A similar effect was observed in another report where chronic lung inflammation in cystic fibrosis (CF) induced by *Pseudomonas aeruginosa* (PA) was targeted by WA. Determination of subunit p65 translocation of NF-κB indicated that WA inhibited PA-induced NF-κB activation in CF. Hence, inhibited inflammatory action [56]. Another pathway for NF-κB activation is through induced nitric oxide synthase (iNOS) and NO production for immunomodulatory and inflammatory disorders. WA repressed these events as was observed in lipopolysaccharide (LPS) activated macrophage like cells (RAW 264.7). It exerted its inhibitory effect on iNOS and NO production by blocking Akt; hence, down-regulated NF-κB [79]

WA also exerted its anti-inflammatory effects by loss of collagen expression and suppression of COX-2 expression (effects produced via P13K/Akt, p38, c-Jun N-terminal kinase (JNK), and through ROS production). WA additionally constrained sulphated proteoglycan and prostaglandin E_2_ (PG E_2_) [80]. The endothelial cell protein receptor (EPCR) has significant importance in inflammation and coagulatory effects. These activities are conferred by shedding of the EPCR that is mediated by TNF-α converting enzyme (TACE). WA obstructed the activity of TNF-α, phorbol-12-myristate-13-acetate (PMA), IL-1b and EPCR shedding induced by cecal ligation and punctures. It also inhibited TACE, PMA induced phosphorylation of p38, extracellular regulated kinase (ERK) ½ and JNK. [20]. Moreover, in another report human islet cell cultures were treated with cytokines and TNF-α to induce inflammation followed by administration of WA to a group. Real time PCR (RT PCR) resulted in lack of expression of inflammatory genes in the cultures treated with WA while the cultures treated with cytokines and TNF-α alone showed significant increase in those genes [64].

#### 3.1.5. Immunomodulation in Cancer

WA demonstrated immunosuppressive effects on B and T lymphocytes and thymocytes of mice [64]. Human T and B lymphocytes form E rosettes and EAC rosettes to induce their immune response, which can be inhibited by WA. In a study, response of host by leukemic xenogenic grafts was studied to assess the functional activity of T lymphocytes. WA affected the immune response against xenogenic grafts and it was concluded that WA reduce proliferation capacity of mitogen activated T lymphocytes [81]. Despite immunosuppressive effect on peripheral lymphocytes, WA activated cytotoxic T (CD8^+^) cells by suppressing assembly of myeloid derived suppressor cells (MDSC). During tumor proliferation, MDSC constrains activation and tumor infiltration of CD8^+^ cells. In vivo study in mice tumors confirmed that WA blocked MDSC accumulation and tumor associated macrophages, reduced tumor volume, and suspended MDSC mediated cytotoxic T cell suppression [82]. This dual role of WA provides benefit of generating immunity particularly against cancer while protecting healthy cells from over-activated cytotoxic T cells by peripheral immunosuppression.

#### 3.1.6. Healing Effects in Bone Metastasis

Metastatic bone disease is the condition where cancer cells originating from organs metastasize to bone causing extensive bone loss. Entry of cancer cells into bone microenvironment triggers a vicious cycle of remodeling [83]. Physiologically, osteoblasts induce production of receptor activator of NF-κB ligand (RANKL) that binds with either RANK receptor to prompt osteoclast differentiation and activation or osteoprotegrin (OPG) receptor to curtail osteoclast activation. Tumor cells down regulate OPG and up regulate RANKL amplifying osteoclast production and bone degradation [84]. It is proposed that damage due to bone metastasis can be prevented by the use of WA since it employs healing effects by simultaneously promoting osteoblastogenesis and inhibition of osteoclastogenesis. It induced the formation of osteoprogenitor cells in marrow and increased expression of osteogenic genes. These effects are conferred by inhibiting proteasomal enzymes and anabolic effects [85]. WA binding with β-catalytic subunit of proteasomal enzymes augments proliferation and activation of osteoblasts by expressing mineralizing genes and transcription factor as well as by suppressing the inflammatory factors. It was found that WA induced bone formation and strengthened the bone matrix by microarchitecture and biomechanical modifications. WA decreased osteoclastin (OCN) and osteoclasts formation by directly decreasing the expression of tartrate resistant acid phosphatase expression (TRAP), RANK and indirectly reducing ratio of OPG/RANKL. Treatment of calvarial osteoblasts with WA resulted in E3 ubiquitin ligase (Smad ubiquitin regulatory factor 2-Smurf2) expression as well as protection of runt-related transcription factor 2 (RunX2) and relevant Smad proteins from degradation. Expression of Smurf2 by TNF-α taken exogenously in primary osteoblasts was also decreased by WA treatment. Furthermore, it inhibited endogenously produced TNF-α and OCN to avoid its turnover effect on bone formation [85]. Synergism of bone regeneration and anti-inflammatory effects of WA shows its importance in the management of bone metastasis.

#### 3.1.7. Anti-Herpetic Effects

Cancer patients are inclined to viral infections owing to immunosuppressive effects of cancer chemotherapy. Herpes simplex virus (HSV) infections are one of the common viruses encountered during cancer chemotherapy [86]. HSV 1 and 2 are dangerous biological warfare responsible for sore throat, flu and some genital diseases. WA inhibits residues, which are crucially important for the proper functioning of DNA polymerase in HSV such as Asn 815, Gln 617, Gln 618, and Tyr 818. WA binds with these residues in a similar way to 4-oxo-DHQ (non nucleosidic viral polymerase inhibitor). It was observed by the molecular dynamics simulations that WA conferred some conformational changes in the binding sites of polymerase consequently acting as a potent ligand binding inhibitor for DNA polymerase in HSV [86]. This effect of WA can be adjunctive to chemotherapy for both prophylaxis and management of HSV infections in cancer patients. 

#### 3.1.8. Anti-Fibrotic Effects

Cancer and tissue fibrosis are interlinked (as one may precede the other). Persistent hepatic and cystic fibrosis lead to hepatocellular carcinoma and digestive tract cancer respectively. It was found in a 20-year nationwide study conducted in the United States that cystic fibrosis increases the risk of gastric, biliary, intestinal, and colon cancers [87]. It was also reported that hepatic fibrosis triggered cirrhosis and hepatocellular carcinoma [88]. On the other hand, radiation therapy of cancer sensitizes some of the patients to the development of myocardial fibrosis. Radiation therapy caused adventitial fibrosis in coronary arteries concerning damage to endothelial cells. This was caused by radiation induced inflammatory vascular damage due to ROS and NF-κB activation [89].

Type 1 collagen is abundantly present in our body and, if excessively produced, causes fibrosis in different organs of the body. Excessive production is due to the stabilization of collagen mRNA. The intermediate filaments are composed of vimentin. Since, WA primarily targets vimentin; therefore, collagen production is inhibited by vimentin disruption. In cuticle and cardiac fibrosis, WA disrupted vimentin filaments and decreased half-life of collagen-a1 and collagen-a2 up to three folds with IC_50_ of 0.5–1.5 mM, thus exhibiting anti-fibrotic effect both in vivo and in vitro. Phosphorylation of transforming growth factor b1 (TGF-b1), Smad3 and collagen gene transcription via TGF-b1 was also inhibited by WA. Anti-fibrotic effect was also seen in hepatic and intestinal fibrosis [90]. This indicates the potential use of WA as preventive therapy in patients undergoing radiation therapy and treatment of fibrosis to avoid cancer development. 

### 3.2. Antiplatelet and Profibrinolytic Effects

WA also possesses anticoagulant, profibrinolytic, and antiplatelet aggregation effects. The profibrinolytic effect was studied by observing the prothrombin time, polymerization of fibrin, partial thromboplastin time, platelet aggregation, plasminogen activator inhibitor type-1 (PAI-1), thrombus formation, and activated factor X (FXa). Human activated umbilical vain endothelial cells (HUVECs) activated by TNF-α were used to evaluate the effect of WA on PAI-1 expression. The effects of WA showed inhibition of fibrin polymerization catalyzed by thrombin, platelet aggregation and thrombus formation induced by FeCl_3_ that significantly prolonged PT and aPTT. It also inhibited FXa and thrombin production. WA prolonged in vivo and ex vivo bleeding time and inhibited TNF-α induced PAI-1. Furthermore, it decreased the PAI/tPA (tissue plasminogen activator) ratio [91].

### 3.3. Antileishmanial Effect

A very remarkable effect of WA was also reported against *Leishmania donovani*. WA, a potent inhibitor of protein kinase C (PKC) caused depolarization of membrane potential Δψ_m_ and generated reactive oxygen species inside *L. donovani* cells. Lower Δψ_m_ increased cytochrome C production inside the cell, consequently activating oligonuceousomal DNA cleavage due to caspase such as proteases generation. It was found that oxidative DNA cleavage was promoted to stabilize topoisomerase-I mediated cleavable complexes (a contributor to DNA fragmentation). It was verified that the apoptotic process can be amplified by stabilizing topoisomerase-I DNA complex. Although the exact process of stabilization is not confirmed as WA was unable to induce stable complex formation with either recombinant topoisomerase-I or extracted DNA from the control cells, but the inhibition of PKC is considered a fundamental step for apoptosis [92]. In another study, immune-prophylactic and therapeutic effects of WA against *L. donovani* infection in hamsters were investigated. Hamsters were fed on the immune stimulatory doses of WA for five days. Afterwards, animals were encountered with *L. donovani* parasites on day 6 and euthanized at day 30 and 45. Post challenge assessment of parasitic clearance depicted that there was a significant increase in Leishmania-specific lymphocyte transformation test (LTT) response as well as ROS, NO and antileishmanial IgG2 levels [93]. This indicates that WA up-regulated NO, ROS, and antibody response in hamsters to eradicate leishmaniasis. As a result, WA becomes a potential candidate for antileishmanial therapy.

### 3.4. Antiadipogenesis Effect

WA has also been reported to affect adipocytes and decrease their viability. It can also promote apoptosis and inhibit adipogenesis. WA in concentration of 1–25 µM for 4 h caused apoptosis in all stages of cell division in pre and post-confluent preadipocytes and mature adipocytes due to phosphorylation of ERK1/2 and altered expression of Bax and Bcl2. WA (0.1–1 µM) reduced peroxisome proliferator-activated receptor γ expression in dose dependent manner and decreased lipid accumulation in cells [58]. It also decreased adipocyte fatty acid binding protein and CCAAT/enhancer binding protein α [58]. 

### 3.5. Antipigmenting Effect

WA also possesses antipigmenting effect with reduced risk of hypo-pigmentation. Hyperpigmentary disorders are inflicted owing to upregulation of endothelin (EDN) 1 and stem cell factor (SCF) in epidermal cells when encountered with UV dependent production of IL-1 and independent release of TNF-α. EDN1 and SCF bind with endothelin B receptor and stem cell growth factor receptor respectively, which stimulate melanocytes and augment tyrosinase mediated synthesis of melanin. It was reported that WA interrupted SCF and EDN1 induced intracellular signaling pathways and abrogated pigmentation in human epidermal equivalents (HEEs). This inhibitory effect on both molecular targets of melanin production cascade abolishes the synergistic cross talk signaling with consequential distinct antipigmenting effect [94]. 

## 4. Summary

Withaferin A being one of the most efficacious withanolides of *W. somnifera* has gained a unique place in therapeutic mainstream. Based on several investigations using different cell lines and animal models, its hidden potential has been unmasked. All of these studies have proclaimed its multidimensional therapeutic role as an anticancer, chemosensitizing, antistress, anti-inflammatory, cardioprotective, antiadipogenic and antipigmenting agent.

Major mechanisms of WA induced anticancer activity are: (i) down-regulation of HPV E6 and E7 oncoprotein expression; (ii) induction of p53 accumulation; (iii) increased p21cip1/waf1 and interaction with PCNA (proliferating cell nuclear antigen) levels; (iv) cell cycle arrest at G2/M phase, associated with modification in cyclin B1, p34, Cdc2, PCNA levels and activation of proteosomal enzymes; (v) STAT3 down-regulation and its phosphorylation at Ser727 and Tyr705; (vi) modification of p53-mediated apoptotic markers (Bcl2, Bax, caspase-3) and reduced PARP expression levels; (vii) decreased lipid peroxidation; (viii) vacuolization of cytoplasm, distention or dissolution of mitochondrial cristae; (ix) disruption of microtubules of mitotic spindles; (x) angiogenesis inhibition by vimentin disruption; and (xi) inhibition of metastasis by blocking extracellular matrix degrading proteins. Other mechanisms are up-regulation of tumor suppressor proteins, FOXO3a- and Bim-dependent apoptosis [49] as well as inhibition of signal transducer and activation of transcription-3 in cancerous cells [50]. 

## 5. Future Prospects

This review shows that based on its preliminary and mechanistic studies, WA can be used to treat multiple ailments. However, the spectrum of its pharmacology activities is required to be defined by detailed research in diseases such as arthritis, hypertension, central nervous system disorders, musculoskeletal disorder, and the hematopoietic system. Two important perspectives that require further research include toxicological evaluation to determine the safety profile and standardization of its pharmacodynamic parameters. Additionally, it is necessary to design formulations of WA that can be administered to the patients to treat particular disease. For this purpose, pharmacokinetic, biodistribution, and formulation design studies must be conducted. We thus recommend advancing WA for clinical trials to make this compound a useful commercial drug with maximum uses and the least side effects.

## Abbreviations

Endothelin (EDN), estrogen receptor α (ERα), cystic fibrosis (CF), heat shock (HS), herpes simplex virus (HSV), human epidermal equivalents (HEEs), lipopolysaccharide (LPS), malignant pleural mesothelioma (MPM), mitogen-activated protein kinase (MAPK NF-κB essential modulator (NEMO), osteoclastin (OCN), osteoprotegrin (OPG), P-glycoproteins (P-gp), poly-(ADP-ribose)-polymerase (PARP), proliferating cell nuclear antigen (PCNA), prostate apoptosis responsive-4 (PAR-4) gene, pseudomonas aeruginosa (PA), myeloid derived suppressor cells (MDSC), reactive oxygen species (ROS), rearranged during transfection of tyrosine kinase (RET), receptor activator nuclear factor κ (RANK), runt-related transcription factor 2 (RunX2), sorafenib (SN), spindle assembly checkpoints (SAC), stem cell factor (SCF), stress activated protein kinase (SAPK), tartrate resistant acid phosphatase expression (TRAP), transgenic mouse mammary tumor virus-neu (MMTV-neu), withaferin A (WA), Withania somnifera (WS).
